# Effects of Living High-Training Low and High on Body Composition and Metabolic Risk Markers in Overweight and Obese Females

**DOI:** 10.1155/2020/3279710

**Published:** 2020-02-11

**Authors:** Huan Gao, Jianfang Xu, Li Zhang, Yingli Lu, Binghong Gao, Lianshi Feng

**Affiliations:** ^1^Shanghai Research Institute of Sports Science, Shanghai, China; ^2^China Institute of Sport Science, Beijing, China; ^3^Shanghai University of Sport, Shanghai, China

## Abstract

This study examined the effects of 4 weeks of living high-training low and high (LHTLH) under moderate hypoxia on body weight, body composition, and metabolic risk markers of overweight and obese females. Nineteen healthy overweight or obese females participated in this study. Participants were assigned to the normoxic training group (NG) or the LHTLH group (HG). The NG participants lived and trained at sea level. The HG participants stayed for approximately 10 hours in a simulated 2300 m normobaric state of hypoxia for six days a week and trained for 2 hours 3 times a week under the same simulated hypoxia. The interventions lasted for 4 weeks. All groups underwent dietary restriction based on resting metabolic rate. The heart rate of the participants was monitored every ten minutes during exercise to ensure that the intensity was in the aerobic range. Compared with the preintervention values, body weight decreased significantly in both the NG and the HG (−8.81 ± 2.09% and −9.09 ± 1.15%, respectively). The fat mass of the arm, leg, trunk, and whole body showed significant reductions in both the NG and the HG, but no significant interaction effect was observed. The percentage of lean soft tissue mass loss in the total body weight loss tended to be lower in the HG (27.61% versus 15.94%, *P*=0.085). Between the NG and the HG, significant interaction effects of serum total cholesterol (−12.66 ± 9.09% versus −0.05 ± 13.36%,) and apolipoprotein A_1_ (−13.66 ± 3.61% versus −5.32 ± 11.07%, *P*=0.042) were observed. A slight increase in serum high-density lipoprotein cholesterol (HDL-C) was observed in the HG (1.12 ± 12.34%) but a decrease was observed in the NG (−11.36 ± 18.91%). The interaction effect of HDL-C between NG and HG exhibited a significant trend (*P*=0.055). No added effects on serum triglycerides (TGs), low-density lipoprotein cholesterol (LDL-C), or APO-B were observed after 4 weeks of LHTLH. In conclusion, 4 weeks of LHTLH combined with dietary restriction could effectively reduce the body weight and body fat mass of overweight and obese females. Compared with training and sleeping under normoxia, no additive benefit of LHTLH on the loss of body weight and body fat mass was exhibited. However, LHTLH may help to relieve the loss of lean soft tissue mass and serum HDL-C.

## 1. Introduction

Obesity has become a global public health concern. The World Health Organization (WHO) estimates that the prevalence of obesity has nearly tripled since 1975; 39% of adults and more than 340 million children and adolescents were overweight or obese in 2016 [1]. In China, 13.2% of children and adolescents aged 7–17 years were obese in 2015 [2]. In 2016, 32.3% of adults were overweight in China [3]. A similar high prevalence was found in many countries [4]. Excess energy intake and low energy expenditure are considered to be the main factors causing obesity [5]. A restricted diet to decrease energy intake has been demonstrated to be effective for weight loss. However, the catabolic effect caused by a reduced energy intake not only reduces fat mass but also causes an undesirable loss of organs and tissues [6]. Aerobic exercise together with energy restriction has been demonstrated as a much effective method to reduce body weight and prevent muscle loss [7, 8].

Losing body weight and body fat mass was commonly observed while climbing or hiking in high altitudes [9–11]. Weight loss occurred even as a result of just staying at a high altitude campus [11, 12]. The body mass of children and adolescents living at moderate altitude was lower than that of those living in the plain [13]. In the rat model, hypoxic exercise led to additive more weight loss and more expression of leptin and leptin receptor in adipose tissue [14, 15]. The above reports suggest that a hypoxic environment may be beneficial for weight loss. Additionally, the beneficial effects of exposure to hypoxia have been shown, such as an enhanced ability to transport oxygen to muscle, the improvement of fat oxidation, and insulin sensitivity [16–18]. Living low-training high (LLTH) which means that just training under hypoxia has been used for obese subjects in several studies. On 3 hours to 4.5 hours per week for 4–8 weeks, an aerobic training intervention under moderate hypoxia showed only slight reductions in body weight [19, 20]. However, Kong et al. reported that 16 hours of exercise in normoxia combined with 6 hours of exercise under moderate hypoxia per week plus dietary restriction composing an intervention lasting for 4 weeks resulted in a 6.9 kg reduction in body weight [21]. Through the above analysis, it suggests that maintaining normal dietary behavior, low exercise volume, and short exposure to hypoxia may be related to the slight reduction of body weight.

Living high-training low (LHTL) has been widely employed to improve aerobic performance in athletes [22–24]. Living high-training low and high (LHTLH) is a modified hypoxic training program. In addition to sleeping in a hypoxic room and training under normal conditions, as in LHTL, some sessions were performed under hypoxic conditions. Several studies on athletes have applied this hypoxic training method [25, 26]. Compared with other methods, the dose of exposure to hypoxia of LHTLH is much higher. Theoretically, LHTLH could result in much higher stress on the body and may be more helpful for encouraging weight loss or regulating metabolic risk factors than other interventions. To our knowledge, no previous study has examined overweight or obese subjects and related physiologic responses employed by LHTLH.

## 2. Materials and Methods

### 2.1. Participants and Experimental Design

According to the suggestion of the Bureau of Disease Prevention and Control of the National Health Commission of the People's Republic of China, the BMI range for overweight was 24.0 to 27.9 kg/m^2^ [27], and individuals with a BMI ≥24 kg/m^2^ whose age was ≥18 years old were involved in the study. For adolescent females whose age was <18 years old, the BMI needed to be higher than the cut-off point for overweight at their age [28]. In addition, participants were excluded if they met anyone from the following criteria: had concomitant renal, hepatic, or cardiovascular disease; was a smoker; had previous hypoxia experiences in the last 3 months; had structured exercise or dieting history in the last 3 months; took drugs to manage body weight; or had any limitations regarding physical activity. For all participants, an exercise electrocardiogram was performed for safety reasons to exclude subjects with cardiac insufficiency. The study program was approved by the Committee for Scientific Research Ethics of China Institute of Sport Science (HE-BS002). Nineteen healthy overweight or obese females were recruited. The residential training camp was organized in a summer vacation. Participants were summited to a 4-week training program and were randomly assigned to the normoxic training group (NG) or the LHTLH training group (HG, Table 1). The purpose and design of this study were explained to every participant. Informed consent was provided by nine juvenile guardians and ten adults themselves before the initiation of the intervention.

### 2.2. Training Protocol

The NG participants lived and trained at sea level. The LHTLH participants stayed in a simulated 2300 m normobaric hypoxia setting from 19:00 to 07:00 every night, combined with 2 hours of aerobic exercise under the same hypoxia environment 3 days per week. Oxygen and carbon dioxide concentrations were persistently monitored by a low oxygen system, while participants remained in a hypoxic laboratory (Low Oxygen System GmbH, Germany). The intervention lasted for 4 weeks performed by 6 days a week and ∼5 hours a day which consisted of four parts involved in one-hour walking or brisk walking in the early morning before breakfast, 2 hours in the morning, one hour in the afternoon, and one-hour walking or brisk walking in the evening after supper. The training intensity corresponded to a 20–40% heart rate reserve (HRR), which was determined by the Karvonen equation [29]. HRR has been used to control exercise intensity in several researches on obesity [30–32]. 40% HRR could be corresponding to about 30% VO_2max_ [33]. Exercise forms consisted of walking, brisk walking, jogging, table tennis, badminton, swimming, aerobic dance, and cycling (Table 2). To ensure that the intensity was in the target range, heart rate and blood oxygen saturation were measured (Nonin 9500 Oximeter, USA) every ten minutes during exercise.

### 2.3. Diet Control

To ensure balanced daily meals, lifestyle education and dietary behavior courses were given by nutritionists before the intervention. The resting metabolic rate was 1605 ± 191 kcal/day, calculated by the Mifflin equation which has been recommended and used for estimating resting energy expenditure in overweight and obese individuals [34]. The diet was given by a nutritional research assistant based on the resting metabolic rate. Three calorie-controlled meals were offered using the same recipe each day in two groups containing macronutrients as follows: protein, 30%; carbohydrates, 50%; and fat, 20%. Breakfast, lunch, and supper accounted for 35%, 40%, and 25% of the total daily energy intake, respectively.

### 2.4. Measurement of Body Composition

Body weight was determined by an electronic scale (TCS-WB-3000, China) and body composition was measured by dual-energy X-ray absorptiometry (GE Lunar Prodigy, soft version 12.2, USA) one day before the beginning and one day after the end of the 4 weeks of intervention.

### 2.5. Measurement of Metabolic Risk Markers

Blood samples were obtained after 12 hours of fasting one day before the beginning and one day after the end of the 4-week trial. Serum free fatty acids (FFAs), total cholesterol (TC), total triglycerides, APO-A_1_, and APO-B were measured with an oxidase assay (Olympus AU640, Japan). The direct method was used to measure high-density lipoprotein cholesterol (HDL-C) and low-density lipoprotein cholesterol (LDL-C) concentrations (Olympus AU640, Japan). The intra-assay coefficient of variation (CV) was less than 5%.

### 2.6. Statistical Analysis

Descriptive statistics are presented as the mean ± standard deviation (SD). Differences between values obtained before and after intervention for the two groups were compared by paired *t*-tests (2-tailed). Two-way ANOVA with repeated measures was employed to determine the effect of the intervention on chosen physiological and biochemical variables. An independent *T*-test was used to analyze the difference in the ratio of body fat mass loss and body lean soft tissue mass loss to body weight loss between the NG and the HG. The level of statistical significance was set at *P* < 0.05, and 0.05< *P* < 0.01 denoted a significant tendency. As an effect size, η^2^ is considered small if *η*^2^ < 0.01, median if *η*^2^ < 0.06, and large if *η*^2^ > 0.14 [35]. Statistical analysis was carried out using SPSS software (Version 17.0, IBM, New York, USA).

## 3. Results

### 3.1. Effect of 4 Weeks of LHTLH Training on Body Weight, Body Fat Mass, and Body Lean Soft Tissue Mass

**Figure 1:**
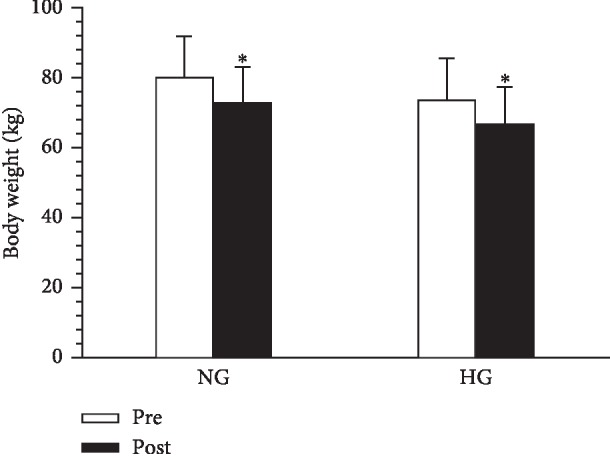
Effect of normoxic and hypoxic exercises on body weight. Data are presented as the mean ± SD. NG, normoxic group. HG, LHTLH group. ^*∗*^*P* < 0.05, pre versus post.

After the 4-week intervention, body weight was significantly reduced in both the NG and the HG (−8.81 ± 2.09% versus −9.09 ± 1.15%). However, no significant interaction effect was observed (Figure 1 (*P*=0.671).

**Figure 2:**
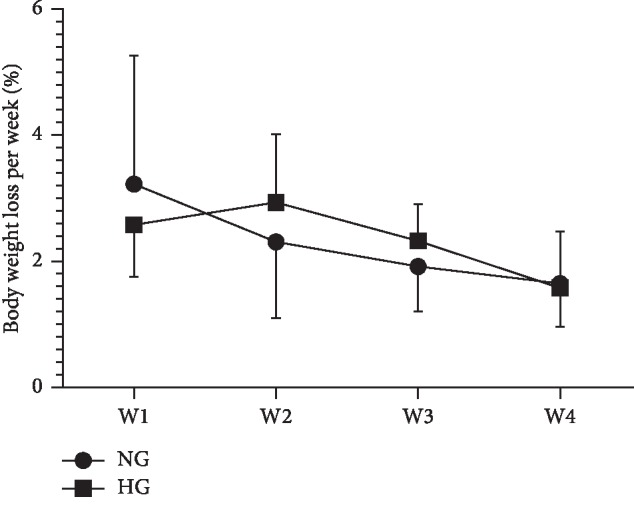
Weekly loss of body weight during normoxic and hypoxic exercise interventions. Data are presented as the mean ± SD. NG, normoxic group. HG, LHTLH group. W1–W4 refers to the 1^st^ week to the 4^th^ week.

No significant interaction effect of weekly loss of body weight was observed after 4 weeks of the intervention in the NG and the HG (Figure 2).

Compared with the preintervention values, the FM and lean soft tissue mass (LSM) of the arm, trunk, and whole body decreased significantly in both the NG and the HG. No significant interaction effect differences between FM and LSM existed (Table 3).

**Figure 3:**
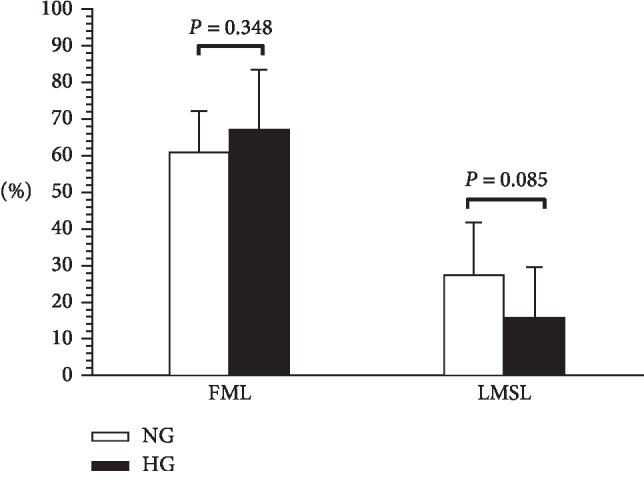
The ratio of the total FM loss (FML) and total LSM loss (LSML) to body weight loss. FML: fat mass loss. LSML: lean soft tissue mass loss. Data are presented as the mean ± SD.

The proportion of the total fat mass loss to total body weight loss of the HG was slightly higher than that of the NG (67.36% versus 61.10%, *P*=0.348). The ratio of the total LSM loss to total body weight loss between the NG and the HG (27.61% versus 15.94%, *P*=0.085) showed a significantly lower trend (Figure 3).

### 3.2. Effect of 4 Weeks of LHTLH on Serum Metabolic Risk Markers

Compared with the preintervention values, serum FFAs increased significantly in both the NG (+71.74%, *P* < 0.01) and the HG (+90.91%, *P* < 0.01), but no interaction effect was observed. Serum TC and APO-A_1_ showed a marked decrease in the NG but not in the HG, and a significant interaction effect was exhibited (*P*=0.040 and 0.042, respectively). No significant changes in serum TGs, LDL-C, or APO-B were observed in either the NG or the HG. A significant loss of serum HDL-C was observed in the NG, but a slight elevation was observed in the HG. A trend toward a significant interaction effect of serum HDL-C was observed between the NG and the HG (*P*=0.055). No significant interaction effect of LDL-C/HDL-C and APO-A_1_/APO-B was observed (Table 4).

## 4. Discussion

In the present study, we observed a significant decrease in body weight in overweight and obese young females after 4 weeks of dietary restriction combined with high-volume aerobic training in both normoxia and simulated hypoxia. The main reduction in body weight consisted of the loss of body fat mass. LHTLH did not show a significant added effect on body weight and body FM. But it exhibited a trend to help attenuate the ratio of LSM loss in the total body weight loss.

The main reasons resulted in the reduction of body weight during climbing or hiking at altitude are manifold and include, but are not limited to, elevated resting metabolic rate, suppressed appetite, increased fluid loss, and impaired gastrointestinal environment [11, 36, 37]. The height of the altitude, the duration of the exposure, the physical activity level, and the energy intake influenced the individual response to hypoxia [38]. However, studies applying hypoxia to help reduce body weight for obesity or overweight are still rare and present inconsistent results. Under moderate hypoxia employed by LLTH with a normal diet, 90 min of low-intensity aerobic training on 3 days every week for 8 weeks resulted in 1.14 kg or 1.3% reductions in body weight and additive improvement exhibited but no weight loss in the control group [20]. Meanwhile, it found that LLTH could help to prevent HDL loss and decrease more cholesterol, triglycerides, and LDL [20]. After 60 min of aerobic running on 3 days per week for 4 weeks without dietary behavior intervention, no significant change and similar reductions of body weight (<2.0%) were observed in both hypoxic group and normoxic group but there was an added decrease of body FM content [19, 39]. Normal diet plus HIIT on 4 days per week for 5 weeks under moderate normobaric hypoxia did not show an additive effect on body weight or body FM but improved more VO_2peak_ [40]. Keeping a usual living lifestyle combined with twice 90 min 65–70% HRmax exercise per week for 8 months, no additive weight loss (∼3.0%) or body FM loss under hypoxic exposure was found [41]. The above studies suggested that, compared with exercise under normoxia, either 4–8 weeks or 8 months of intervention and normal diet combined with training under hypoxia caused a similar and slight reduction in body weight.

Dietary restriction to decrease energy intake and longer exposure duration to hypoxia may be important to lose weight for obesity. In another study, a dietary restriction was added to a regular exercise intervention. It was reported that 4 weeks of a low-calorie diet plus exercise 6 h/week in 3000 m normobaric hypoxic conditions in addition to exercise 16 h/week in normoxic conditions led to significantly more weight loss than that observed in the normoxic group (−7.0% versus −4.2%) [21]. LHTL also reduced more body weight in Yang's report [42]. In our study, the daily energy intake met the resting metabolic needs which is 3- to 4-fold higher than that used in Kong's research [21]; however, more weight loss was observed. One of the reasons may be due to the much greater energy expenditure resulted from the higher amount of exercise and much longer duration exposure to hypoxia. Although there was no additional effect on both weight loss and total fat mass loss or fat mass loss in other regions, a lower percentage of total LSM loss in the total weight loss was exhibited after LHTLH. The underlying mechanism is not clear. This finding may be related to the lower absolute exercise workload [20] and partly due to the increase in blood perfusion and the elevated number of capillaries in skeletal muscle after hypoxic training [43, 44].

The concentration of serum TC is still considered to be the gold standard for the estimation of cardiovascular disease (CVD) risk [45, 46]. Higher serum TC and LDL-C have been reported to be associated with a higher incidence of CVD [47]. At the age of 18–34 years, each 1 mmol/L higher TC increased mortality by 14% [48]. High levels of TC and LDL-C occurred in female adolescents in recent years [49]. A combination of low energy diet and aerobic interval training has been recommended as an effective prescription to coronary artery disease (CAD) patients [50]. Hypoxia could activate the transcription of peroxisome proliferator activated receptor-*γ* coactivator-1*α* (PGC-1*α*) by coactivating the PPAR through hypoxia-inducible factor (HIF) pathway to regulate the fatty acid oxidation [51]. From the theoretical point of view, exercise combined with hypoxic exposure could be much helpful to reduce more blood lipids. For prediabetes patients, it has also been repeated breath intermittent hypoxia (12% O_2_) could decrease serum glucose, cholesterol, and LDL [52, 53]. Applying hypoxic training to improve blood lipids in individuals with obesity is one of the main expectations. Among healthy subjects, 3 sessions per week for 4 weeks of intermittent hypoxic training did not show additive effects on blood TC, LDL-C, or HDL-C compared with training under normoxia [54]. Most previous publications addressing overweight or obesity reported that training under hypoxia has not additive benefits to the serum lipid profiles compared with training under normoxia [19, 20, 40]. After 10 days of moderate-altitude walking, the TC and LDL-C of metabolic syndrome subjects decreased significantly but with almost the same change as that of the low-altitude control group [55]. In the present study, we also did not observe the added effects of LHTLH on serum TG, LDL-C, and APO-B; however, we observed the interaction effects of LHTLH on serum TC and APO-A_1_. LHTLH could prevent the loss of Apo-A_1_ during this intervention program. It is in line with the slight increase of serum HDL-C in HG. This result is similar to Netzer's finding [20].

HDL-C plays an important role in cardiovascular health, such as increasing angiogenesis [56], and protective effects of CAD [57]. Endurance exercise could elevate plasma HDL-C [58]. A higher amount of exercise always resulted in greater improvements in HDL-C [59]. But low-fat diet decreased HDL-C due to the reduced fat intake [60, 61]. In the present study, combining dietary restriction with endurance training together, we found a significant decrease of HDL-C in the normoxic training group but a slight increase in the hypoxic training group. One possible explanation is that, under exposure to hypoxia, the upregulated HIF could increase the expression of the lipoprotein lipase gene through PPAR*γ*1 to prevent the loss of HDL-C [62]. Furthermore, we observed the higher magnitude of elevated serum FFA after LHTHL in the fasting state even with a decrease in serum TG, which is concordant with Mahat's report [63]. In previous studies, it has been demonstrated that the substrate shifts from carbohydrates to lipids for the energy source during the postexercise recovery period [64]. The possible mechanism is that the FFAs released by the elevation of fat lipolysis are not utilized directly for the TG synthesis in the liver but enter the muscle or other organs to be an energy substrate. Hypoxia could amplify this effect. Future studies are needed to confirm and explore the potential mechanism.

From these results, it is still difficult to conclude that hypoxic exercise provides additional benefits for losing weight or improving lipid metabolism in the context of obesity. The normal levels of lipid variables among our participants and the training effect caused by the high-volume aerobic exercise resulted in a deeper stimulation, which exceeded that of the hypoxic effect and may be responsible for the similar changes observed [65]. Great individual differences of lipid response to dietary restriction among humans may also be partly responsible for this difference [66].

This study has several inherent limitations. Relatively small sample size and uneven distribution of age between two treatment groups existed in our project. Therefore, the differences in body weight, body composition, and lipid profiles need to be interpreted with caution. During the intervention, we used the same recipe, employed nutrition assistant to keep the diet energy intake similarly, and measured the heart rate every ten to fifteen minutes to keep the exercise intensity in the aerobic range in both two groups but we did not collect the data as a statistical parameter. In our investigation, we just measure the variable before and after 4 weeks of intervention. More research is needed to explore how long the intervention effect can be lasted.

## 5. Conclusions

Four weeks of living high-training low and high combined with dietary restriction could effectively reduce the body weight and body fat mass of overweight and obese females, but no additional benefits were exhibited compared with those associated with normoxic training. However, the living high-training low and high protocol combined with dietary restriction may help to prevent the loss of LSM and serum HDL-C.

## Figures and Tables

**Table 1:** Subject characteristics.

	NG	HG
Subjects, *n*	9	10
Age, years	16.61 ± 1.96	19.30 ± 1.92
BMI (kg/m^2^)	28.88 ± 4.08	27.97 ± 3.58

**Table 2:** The daily composition of the training load.

Physical activities	Duration (min/d)	Intensity
Walking or brisk walking	120	20–40% HRR velocity<6 km/h
Jogging	40	20–30% HRR velocity <6 km/h
Swimming or cycling	40	30–40% HRR
Table tennis	40	20–40% HRR
Badminton	40	20–40% HRR
Aerobic dance	30	20–40% HRR

**Table 3:** Changes in body FM and LSM before and after 4 weeks of the intervention.

Variable	Pre	Post	*P* value (interaction effect)	*η* ^2^ (effect size)
Arm FM, kg				
NG	3.85 ± 1.14	3.28 ± 0.98^$^	0.352	0.051
HG	3.34 ± 0.82	2.88 ± 0.78^$^		
Leg FM, kg				
NG	11.56 ± 2.76	10.16 ± 2.50^$^	0.092	0.158
HG	10.83 ± 2.49	9.12 ± 2.31^$^		
Trunk FM, kg				
NG	17.86 ± 3.86	15.59 ± 3.30^$^	0.903	0.001
HG	15.66 ± 3.58	13.45 ± 3.35^$^		
Total FM, kg				
NG	34.27 ± 7.12	29.98 ± 6.19^$^	0.771	0.005
HG	30.80 ± 6.31	26.35 ± 5.76^$^		
Arm LSM, kg				
NG	4.23 ± 0.82	4.13 ± 0.74^$^	0.139	0.124
HG	3.73 ± 0.87	3.71 ± 0.73		
Leg LSM, kg				
NG	15.05 ± 1.83	14.60 ± 1.97^$^	0.659	0.112
HG	14.13 ± 2.65	13.77 ± 2.38^$^		
Trunk LSM, kg				
NG	19.84 ± 2.87	18.46 ± 2.40^$^	0.108	0.145
HG	18.01 ± 3.17	17.31 ± 3.20^$^		
Total LSM, kg				
NG	42.57 ± 5.21	40.53 ± 4.86^$^	0.111	0.142
HG	39.22 ± 6.88	38.07 ± 6.51^$^		

Pre, preintervention. Post, postintervention. ^$^*P* < 0.05, pre versus post. NG: normoxic training group; HG: hypoxic training group; FM: fat mass; LSM: lean soft tissue mass.

**Table 4:** Changes in the serum lipid profile after 4 weeks of the intervention.

Variable	Pre	Post	*P* value (interaction effect)	*η* ^2^ (effect size)
FFA, mmol/L				
NG	0.46 ± 0.11	0.79 ± 0.30^$^	0.206	0.098
HG	0.55 ± 0.20	1.05 ± 0.26^$^		
TGs, mmol/L				
NG	1.05 ± 0.33	0.85 ± 0.18	0.554	0.021
HG	0.97 ± 0.30	0.85 ± 0.13		
TC, mmol/L				
NG	4.49 ± 0.79	3.87 ± 0.43^$^	0.040	0.225
HG	4.37 ± 1.16	4.32 ± 0.99		
LDL-C, mmol/L				
NG	2.50 ± 0.75	2.34 ± 0.34	0.545	0.022
HG	2.50 ± 0.93	2.52 ± 0.75		
HDL-C, mmol/L				
NG	1.29 ± 0.22	1.11 ± 0.19^&^	0.055	0.200
HG	1.27 ± 0.20	1.30 ± 0.33		
LDL-C/HDL-C				
NG	1.96 ± 0.53	2.16 ± 0.50	0.305	0.062
HG	2.04 ± 0.98	2.04 ± 0.82		
APO-A1, g/L				
NG	1.16 ± 0.16	1.00 ± 0.13^$^	0.042	0.221
HG	1.23 ± 0.14	1.17 ± 0.23		
APO-B, g/L				
NG	0.72 ± 0.16	0.67 ± 0.10	0.203	0.094
HG	0.70 ± 0.26	0.71 ± 0.19		
APO-A1/APO-B				
NG	1.69 ± 0.42	1.54 ± 0.38^$^	0.566	0.020
HG	1.90 ± 0.50	1.71 ± 0.43^$^		

Pre, preintervention. Post, postintervention. ^$^*P* < 0.05; ^&^0.05 < *P* < 0.1, pre versus post. NG: normoxic group; HG: hypoxic group; FFA: free fatty acid; TC: total cholesterol; TGs: total triglycerides; LDL-C: low-density lipoprotein cholesterol; HDL-C: high-density lipoprotein cholesterol; APO-A1 and APO-B: apolipoprotein A1 and apolipoprotein B.

## Data Availability

The data used to support the findings of this study are available from the corresponding author upon request.
